# COPD uncovered: an international survey on the impact of chronic obstructive pulmonary disease [COPD] on a working age population

**DOI:** 10.1186/1471-2458-11-612

**Published:** 2011-08-01

**Authors:** Monica J Fletcher, Jane Upton, Judith Taylor-Fishwick, Sonia A Buist, Christine Jenkins, John Hutton, Neil Barnes, Thys Van Der Molen, John W Walsh, Paul Jones, Samantha Walker

**Affiliations:** 1 Education for Health, Warwick, UK; 2 National Respiratory Training Center, Virginia, USA; 3 Oregon Health & Science University, Portland, USA; 4 Woolcock Institute of Medical Research, Sydney, Australia; 5 York Health Economics Consortium, York, UK; 6 The London Chest Hospital, Medical and Emergency Directorate, London; 7 University Medical Center Groningen, Department of General Practice, Netherlands; 8 Alpha-1 Foundation, Florida, USA; 9 St George's University of London, London, UK

## Abstract

**Background:**

Approximately 210 million people are estimated to have chronic obstructive pulmonary disease [COPD] worldwide. The burden of disease is known to be high, though less is known about those of a younger age. The aim of this study was to investigate the wider personal, economic and societal burden of COPD on a cross country working-age cohort.

**Methods:**

A cross-country [Brazil, China, Germany, Turkey, US, UK] cross-sectional survey methodology was utilised to answer the research questions. 2426 participants aged 45-67 recruited via a number of recruitment methods specific to each country completed the full survey. Inclusion criteria were a recalled physician diagnosis of COPD, a smoking history of > 10 pack years and the use of COPD medications in the previous 3 months prior to questioning. The survey included items from the validated Work Productivity and Activity Impairment [WPAI] scale and the EuroQoL 5 Dimension [EQ-5D] scale. Disease severity was measured using the 5-point MRC [Medical Research Council] dyspnoea scale as a surrogate measure.

**Results:**

64% had either moderate [n = 1012] or severe [n = 521] COPD, although this varied by country. 75% of the cohort reported at least one comorbid condition. Quality of life declined with severity of illness [mild, mean EQ-5D score = 0.84; moderate 0.58; severe 0.41]. The annual cost of healthcare utilisation [excluding treatment costs and diagnostic tests] per individual was estimated to be $2,364 [£1,500]. For those remaining in active employment [n: 677]: lost time from work cost the individual an average of $880 [£556] per annum and lifetime losses of $7,365 [£4,661] amounting to $596,000 [£377,000] for the cohort. 447 [~40%] of the working population had retired prematurely because of COPD incurring individual estimated lifetime income losses of $316,000 [£200,000] or a combined total of $141 m [£89.6 m]. As the mean age of retirees was 58.3 and average time since retirement was 4 years, this suggests the average age of retirement is around 54. This would mean a high societal and economic impact in all study countries, particularly where typical state retirement ages are higher, for example in Brazil, Germany and the UK [65] and the US [65,66,67], compared to Turkey [58 for women, 60 for men] and China [60].

**Conclusions:**

Although generalisation across a broader COPD population is limited due to the varied participant recruitment methods, these data nevertheless suggest that COPD has significant personal, economic and societal burden on working age people. Further efforts to improve COPD diagnosis and management are required.

## Background

Chronic Obstructive Pulmonary Disease [COPD] is one of the world's most common non-communicable health problems [1]. Approximately 210 million people are known to have the condition [2], although the true prevalence may well be higher due to under-diagnosis [3]. By 2020, COPD is predicted to become the third leading cause of death worldwide [4]. Prevalence is increasing both in developing and developed countries as a result of worldwide tobacco consumption [5,6], environmental exposures such as biomass fuel smoke [7] and the growing elderly population [8]. It is recognised that comorbid diseases commonly occur with COPD [9-11]. Although COPD is considered to be a disease of later years, estimates suggest that 50% of those with COPD are less than 65 years old [12,13], many of whom are likely to be in paid employment.

Although there is a wealth of epidemiological data on the global impact of COPD, data on its economic impact are limited, particularly in terms of how the condition directly affects younger sufferers' ability to work and maintain active productive lives. The World Health Organisation [WHO] has estimated that globally COPD results in an annual loss of productivity of 27,700 years [measured by disability adjusted life years [DALYs] [14]. This estimation places COPD eleventh as a worldwide cause of disease burden; by 2030 [15] it is predicted to be seventh highest.

It is important that we understand the true costs of chronic diseases such as COPD to inform healthcare policy and to target resources effectively [16]. Several studies in the US, UK and Europe have attempted to highlight the financial burden of COPD by estimating the direct costs of health care utilization [17,18] or lost productivity [19,20]. However, direct costs only account for a proportion of cost, and studies to date have not captured the full extent of the economic burden in terms of impact on younger individuals, their families and society as a whole.

### Objectives

This cross-country cross-sectional survey aimed to expand current understanding of the impact of COPD and to demonstrate its wider potential costs in a working age population. It was designed as a hypothesis-generating exercise to identify the factors likely to influence the economic burden of the disease, which can be the subject of further research. Here we describe the observed effects of COPD on: income, employment, work productivity, healthcare utilization and quality of life.

## Methods

### Study design and respondents

The cross-sectional survey was conducted between July and September 2009 in Brazil, China, Germany, Turkey, UK and US. A contract research organisation [CRO] identified, recruited and interviewed respondents following a protocol.

The aim was to recruit a broad mix of respondents; representing wide demographics [including working status] and with a range of disease severity. A mixed methods approach to recruitment was therefore used dependent on country-specific factors. These recruitment methods are described in Table 1.

**Table 1:** Recruitment and questioning procedure

Country	Recruitment procedure	Interview method
Brazil	Random selection from a list obtained from	Telephone*^1 ^and
	COPD patient associations [telephone	face-to-face*^2^
	interviews], in front of major hospitals, and	
	within a variety of public areas with high	
	footfall rates, including parks and squares	
	[face-to-face approach]. The towns and	
	cities included São Paulo, Rio de Janeiro,	
	Belo Horizonte, Fortaleza, Porto Alegre,	
	Curitiba, Vitoria, Recife, and represent most	
	regions within the country.	
		
China	Shanghai was selected as a metropolitan	Face-to-face*^3^
	city. It was divided into 300 regions. 40	
	regions were randomly selected [the target	
	sample size for each region was 10	
	participants]. The interviewer selected the	
	starting point using a random method,	
	usually the crossing point of two major	
	roads. The interviewer then selected every	
	fifth property using the right-hand rule	
	[always turn to the right for the next potential	
	respondent]-side of the particular street	
		
Germany	Random selection from a list obtained from	Telephone*^1^
	fieldwork, recruitment agencies and the	
	database at the CRO, self-help groups,	
	COPD patient associations	
	www.lungenemphysem-copd.de, deustsche-	
	empysemgruppe.de] and panelists of online	
	institutes	
		
Turkey	Two representative cities were selected	Face-to-face*^3^
	from each of the six geographical regions:	
	Marmara [Istanbul and Bursa], Aegean	
	[Izmir and Manisa], Central Anatolia [Ankara	
	and Kayseri], Black Sea [Samsun and	
	Trabzon], Mediterranean [Adana and	
	Antalya] and Eastern Turkey [Diyarbakir and	
	Elazig]. The districts and the streets within	
	each city were randomly selected. A	
	stratified random sample of buildings was	
	used. The interviewers adopted a walking	
	rule for each street, approaching the	
	occupant at every fourth property on the	
	right-hand-side. All work places were also	
	contacted within each of these buildings.	
		
UK	6-8 participants were recruited from 40	Telephone*^1^
	general practices and 25 pharmacies [at	
	least one per major town/city] and invited to	
	contact the researchers. The British Lung	
	Foundation regional groups and local	
	'Breathe Easy' groups also randomly	
	selected members.	
		
US	Random selection from a known list of	Telephone*^1^
	people diagnosed with: COPD obtained	
	from a market research organization	

*^1^Interviews were conducted at a variety of times between 09:00 and 21:00, Monday-Sunday.

*^2 ^Interviews were conducted in public places, including parks and outside a hospital.

*^3 ^Door-to-door interviewing employing the stated sampling criteria.

### Inclusion Criteria

Respondents were eligible if they were 45-67 years, reported a physician diagnosis of COPD, Chronic Obstructive Airways Disease [COAD], Chronic Obstructive Lung Disease [COLD], emphysema, chronic bronchitis or alpha 1-antitrypsin deficiency [A1AD], and had been prescribed respiratory medication during the preceding three months. With the exception of those with A1AD, respondents had to be current or ex-smokers with a minimal history of ten pack years. Smoking history was used to enhance the accuracy of identification of those with COPD, as in Western countries 80-90% of people with COPD are likely to be current or past smokers [18]. In developing countries, environmental factors play a significant role in the aetiology of the disease [7,21]. As a result, respondents in Brazil and China who did not have a minimum cigarette pack year history but otherwise met the inclusion criteria were included if they were at risk of COPD via biomass exposure. This risk was defined as having an indoor open fire and using solid fuel as a primary means of cooking or heating for more than 6 months across the lifetime.

### Questionnaires

Interviews were conducted using a structured survey incorporating:

• Clinical and demographic data

• Recall of physician-diagnosed comorbidities: adapted from a pre-determined list of conditions [17]. The list included the following comorbidities: arthritis, asthma, cancer or tumour, anxiety, depression, diabetes, cardiovascular disease, hypertension, other lung conditions [invitation to specify which], and none.

• Disease severity classified as mild [1,2], moderate [3,4] or severe [5] using the Medical Research Council dyspnoea scale modified by Bestall et al [22].

• The amount of health care utilised within the last month, including GP, outpatient, inpatient utilisation, emergency hospital services and pulmonary rehabilitation.

• Quality of life was measured using the validated, generic, preference-based EuroQoL Group 5 Dimension [EQ-5D] self-report questionnaire [23], which has been applied to a wide range of health conditions. The EQ-5D contains 5 components; 'mobility', 'self-care', 'usual activities', 'pain/discomfort' and 'anxiety/depression.' Each component is scored as either: 'no problems' [level 1], 'some problems' [level 2] or 'severe problems' [level 3], defining the patient's current health state. Each possible health state has been valued, from a societal perspective, on a scale of 1 representing full health and 0 for dead [range -0.594 to 1 where negative values are valued as worse than dead] using preference-based methods by a sample drawn from the general population. The self-defined health state of the patient is thus linked to the social utility value in the two-stage process. The average values for the respondents were compared to age-adjusted population values for those countries where normative data were available [Germany, US and UK] [24].

A further dimension measured 'health state today' on a visual analogue scale [VAS] described to the respondent, where 0 represented 'worst imaginable state', 100 being 'best imaginable state'. The results are then presented on a 0 to 1 scale, whereby a score of 1 represents the best health state imaginable and 0 represents a health state equivalent to being dead. This gives a patient valuation of the health state [albeit non-preference-based] as opposed to the societal value from the questionnaire and matrix.

Impact on work in the preceding 7 days was estimated using the Work Productivity and Activity Impairment Questionnaire: Specific Health problem V2.0 [WPAI: SHP] [25]. To assess the overall impact on productivity, the WPAI generates 4 scores: absenteeism [hours of work missed due to illness], presenteeism [impact of illness on productivity while at work], work productivity loss [combined impact of absenteeism and presenteeism] and activity impairment [regular activities]. The estimates of reduced work input were valued using average earnings for each country in the absence of detailed information on occupational categories. We used the human capital approach to measure productivity losses as it includes all productive time lost by those of working age [26].

The losses to society from premature retirement were estimated by calculating the average annual earnings for the number of years of full-time work lost. We used a pragmatic approach, in the absence of reliable data on friction periods for all countries. Total productivity losses were estimated and sensitivity analysis applied to show the potential reduction in social effect if indeed absent workers were easily replaced.

Additional indicators of burden were identified during patient focus groups and used to generate further questions. These included the economic impact on carers and family, the care needs of patients, work history, social activities, healthcare utilisation, daily activities and future aspirations. Questions were then piloted in face-to-face interviews with COPD patients and amended as necessary. The final survey was piloted in the UK by telephone and in China using door-to-door interviewing. Surveys were translated into the main language of each country, back translated and revised as needed. Final survey interviews were conducted in the main language of each country.

### Ethics

Ethical approval was not deemed necessary following careful consideration by Education for Health internal governance committee and the international steering group. The survey took place among the general public and the data was collected and the dataset generated via a contract market research company. Participation in the study was voluntary and data was collected anonymously. Respondents were informed that their opinions would remain confidential and the data would be collated and presented in an aggregated and anonymous format. All respondents provided informed consent prior to participation.

The study was undertaken in accordance with market research standards and Codes of conduct including ISO 20252 [http://www.mrs.org.uk/standards/other.htm], Market Research Society Code of Conduct [http://www.mrs.org.uk/standards/codeconduct.htm], EPHMRA [http://www.ephmra.org/professional-standards.aspx] ESOMAR [http://www.esomar.org/]. This binds the CRO to all applicable laws protecting personal data.

### Health economic data

The financial impact of COPD was estimated for each country as the annual loss of income resulting from work impairment and projected to lifetime losses using expected working life data. The sample total was expressed in US dollars using purchasing power parities [PPP]. The pound sterling figures were calculated from a simple exchange rate conversion. [$1 = £0.63].

The underlying assumptions were a retirement age of 65 years, an average hourly wage from national income statistics and in the absence of long term data, that the number of working hours lost per week due to COPD would remain constant over a working lifetime. Due to the progressive nature of the condition these could be conservative assumptions as the increasing severity of the disease is likely to lead to increasing impact on work productivity before forced retirement. This will partly be offset by the use of average wage rates when COPD patients may earn less than the average. The customary retirement age [in years] in the study countries at the time of the study was as follows: Brazil, Germany and the UK, 65; US, 65-67; China, 60 and Turkey, 58[F] & 60 [M]. A sensitivity analysis was carried out for those retiring over 65 years assuming a standard age of 67 years.

Annual income loss was calculated using the following formula: number of respondents × % with work impairment × average hours lost per week × hourly wage × 52. The lifetime loss formula: 65 minus actual age of retirement × annual loss discounted at 3.5% per annum.

Potential loss of income was calculated for carers. In the absence of detailed information we made the following assumptions about carers' ability to participate in the workforce: those who provided constant care were assumed to be out of the paid labour force; carers who provided care for part of each day were assumed to miss a full working week; those providing care on 2-3 days; half a working week; and those providing care on one day, one fifth of a working week. Illustrative figures are presented using the UK as a reference point, assuming an annual average wage of £23,937 [$ = 37,820].

To evaluate the impact of COPD on healthcare costs the observed rates of healthcare resource use were multiplied by unit costs in local currencies, which were available for all countries except Brazil. The aggregate impact across all countries was expressed in US$ by applying PPPs to the local totals [27-32] [personal communication for Turkish PPPs]. It was not possible to cost pulmonary rehabilitation due to the wide variation in service provision across the study countries in terms of the number and content of sessions.

### Sample size and data analysis

Based on a previous international survey of COPD patients [17], we selected a sample size of 400 respondents in each of the study countries.

Descriptive statistics [mean, standard deviation or standard error and confidence intervals [95%] were used to report demographic, clinical and epidemiological data and categorised into three age bands: 45-54, 55-64 and 65-67 years. Individual country data were pooled to reflect the international burden of the disease. Data were analysed using STATA v9.

## Results

**Figure 1:**
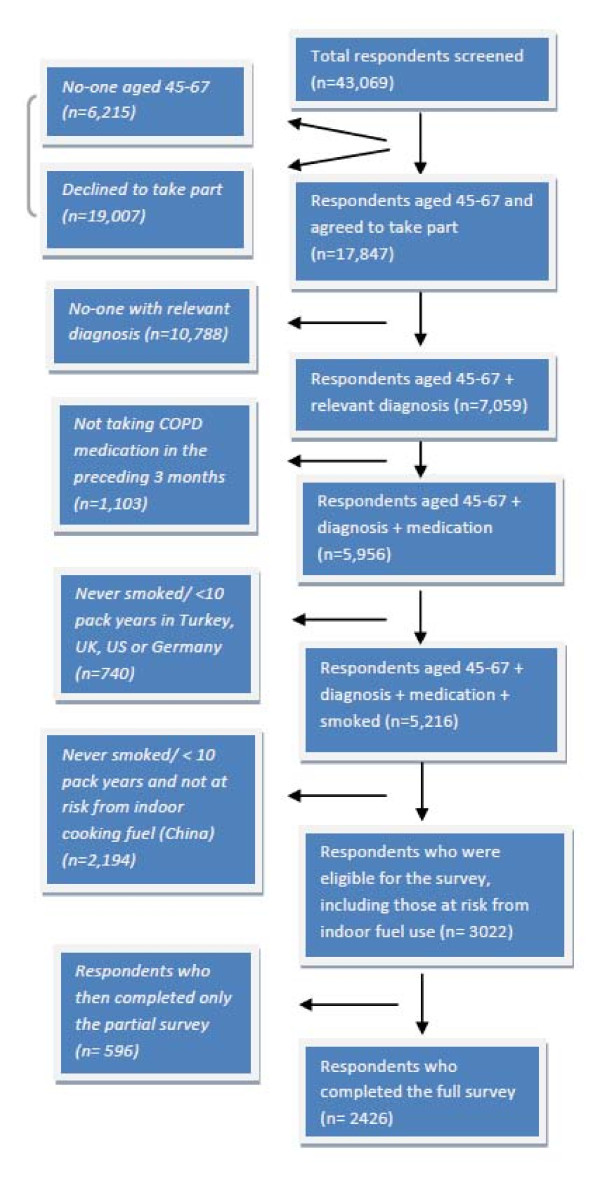
**CONSORT diagram showing recruitment of study participants**. The CONSORT diagram shows the number of people involved at each stage of the survey recruitment process, according to the study criteria. Where a particular individual did not fulfil a particular criterion, they were not included in the study, and where such instances occurred, this is reflected along the left hand side of the figure. The right hand side reflects those included at each stage of the process, including the number of people [n]. The final figure included the respondents completing the full survey.

43,069 respondents were initially approached. Whilst 19,007 declined to partake, others did not meet the age; diagnosis, medication or smoking criteria [see Figure 1].

3022 then fulfilled the full inclusion criteria. 596 surveys were not included due to non-completion, leaving a total of 2426 in the study.

### Clinical and demographic data

**Figure 2:**
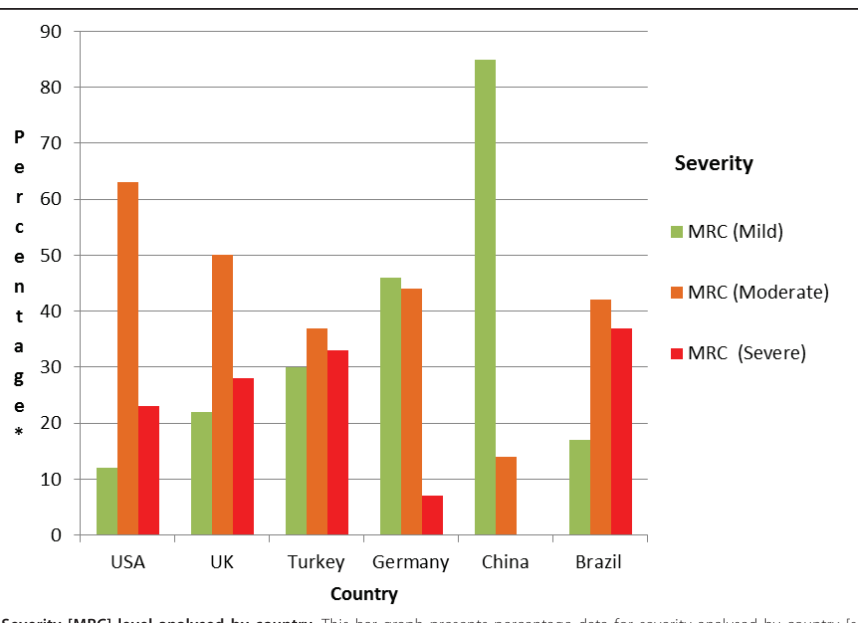
**Severity [MRC] level analysed by country**. This bar graph presents percentage data for severity analysed by country [severity measured using the MRC dyspnoea scale]. *percentage of respondents according to severity level as measured using the Medical Research Council [MRC] dyspnoea scale. There were no respondents in China who reported severe illness.

A similar number of respondents were recruited in each country and the pooled dataset represent a broad mix of ages and disease severity and respondents were evenly distributed in relation to gender [table 2]. Levels of disease severity were widely distributed across the cohort, and there was substantial variation by country [Figure 2].

**Table 2:** Clinical and demographic data of respondents

Group [%]	Sub-group	**N **[2,426]	Males
Country	Brazil	408 [17]	148 [36]
	USA	404 [17]	168 [42]
	Germany	400 [16]	167 [42]
	UK	400 [16]	208 [52]
	China	398 [16]	283 [71]
	Turkey	416 [17]	206 [49]
Total		2426	1180 [49]
Age	45-54	1029 [42]	
	55-64	971 [40]	
	65-67	426 [18]	
Working Status		n [%]	Males [%]
n = 2400 [%]	Working	710 [29]	360 [51]
	Not working	1243 [51]	599 [48]
	Retired early due to COPD*	447 [18]	208 [47]
	Missing	26 [1]	
Severity: [MRC Dyspnoea Scale]	Mild [1, 2]	849 [35]	
	Moderate [3, 4]	1,012 [42]	
	Severe [5]	521 [22]	
	Missing data	44 [2]	
Ever smoked on a daily basis	Yes	2311 [95]	
	No	115 [5]	
Currently smoke	Yes	1366 [59]	
	No	1060 [41]	
	Mean Pack Years	38	
Number of co-morbidities by number of respondents [n = 2,404]	0	595 [25]	
	1	753 [31]	
	2	443 [18]	
	3	265 [11]	
	> 4	348 [14]	
	Missing data	22 [1]	

*early retirees

Mean EQ-5D score declined with increasing age and more markedly with increasing severity of disease [see Table 3]. These comparisons were both highly significant using independent t-tests. This pattern was observed both for the utility scores calculated from the EQ-5D questions, which reflected the societal perspective, and the direct patient scores recorded on the VAS [Table 4].

**Table 3:** EQ-5D scores presented by sex, severity and age

	Males *n: 1,178*				Females *n: 1,243*		
Mean EQ-5D	0.678		s.e	0.009	0.596	s.e	0.009
	MRC scale		Mean	s.e	n	[%]	
			EQ-				
			5D				
							
EQ-5D by severity	Mild:		0.836	0.007	846	[35]	
	Moderate:		0.579	0.009	1011	[42]	
	Severe:		0.409	0.015	521	[22]	
	Missing data				48	[1]	
	Total		0.636	0.007	2426	[100]	
EQ-5D by age:	Age						
	Band	Mean	EQ-5D	s.e.	n	[%]	
	Norm						
	**						
	45-54	0.686	0.85	[0.010]	1028	[42]	
	55-64	0.605	0.80	[0.011]	969	[40]	
	65-68	0.585	0.79*	[0.148]	424	[18]	
	Missing data				5		

Estimated from data for 60-69 age group

Based on UK age norms

**Table 4:** EQ-5D Scores from the Visual Analogue Scale [VAS] [scale]^[23]^

		n	EQ-5D VAS [se]
Country	Brazil	408	0.623 [0.010]
	China	385	0.799 [0.005]
	Germany	400	0.597 [0.011]
	Turkey	416	0.543 [0.009]
	UK	399	0.511 [0.010]
	USA	404	0.544 [0.011]
			
	*Total [N]*	2,412	0.601 [0.004]
Severity	Mild	837	0.733 [0.005]
	Moderate	1,011	0.561 [0.006]
	Severe	521	0.459 [0.009]
Age	45-54	1,024	0.640 [0.006]
	55-64	963	0.583 [0.007]
	65-74	425	0.549 [0.001]

The EQ-5D scale is scored 0 [worst imaginable state] to 100 [best imaginable health state] and is presented on a 0 to 1 scale. A lower EQ-5D VAS score indicates worse quality of life

Compared with age-related population values for the UK general population, EQ-5D values in the cohort were lower reflecting poorer quality of life [see Table 3]. Comparison with EQ5D norms from the US and Germany demonstrated a similar relationship [24]. The mean EQ5D score for the total cohort was significantly lower for females [p = 0.000].

### Comorbidities

1,809 [75%] respondents stated they had ≥1 comorbid condition [median 2, range 1-8]. The most commonly reported conditions were hypertension, asthma, arthritis, anxiety, depression and diabetes. Respondents with mild COPD reported less comorbidity than those with more severe disease [Table 5].

**Table 5:** Comorbidities according to disease severity

	Mild [MRC*** 1-2****]	Moderate [MRC 3-4]	Severity [MRC 5]
	[n = 849]	[n = 1012]	[n = 521]
Zero comorbidities	44% [376]	15% [152]	14% [71]
[n = 595] [%**]			
1-2 comorbidities	46% [391]	53% [533]	49% [255]
[n = 1196]			
≥3 comorbidities	10% [82]	32% [327]	37% [195]
[n = 613]			

*2382 reported disease severity [44 = missing data from cohort of 2426]

**The percentage with a particular level of disease and number of co-morbidities within each disease level

***= Medical Research Council

****1-2 = mild severity of disease, 3-4 = moderate severity of disease, 5 = severe disease

As the number of comorbidities increased quality of life decreased accordingly [mean EQ 5D score 0.807 SD [0.01] with 0 comorbidities; 0.661 SD [0.009] with 1-2 comorbidities; and 0.418 [0.014] with ≥3 comorbidities]. The average difference in EQ-5D scores between participants with no comorbidities and those with 1 or 2 comorbidities was 18%, and for those with more than 2, the difference was 48%. Those retiring early due to COPD had on average 2.5 [mean] comorbidities compared to 1.1 in those still in active employment.

### Health care utilisation

**Figure 3:**
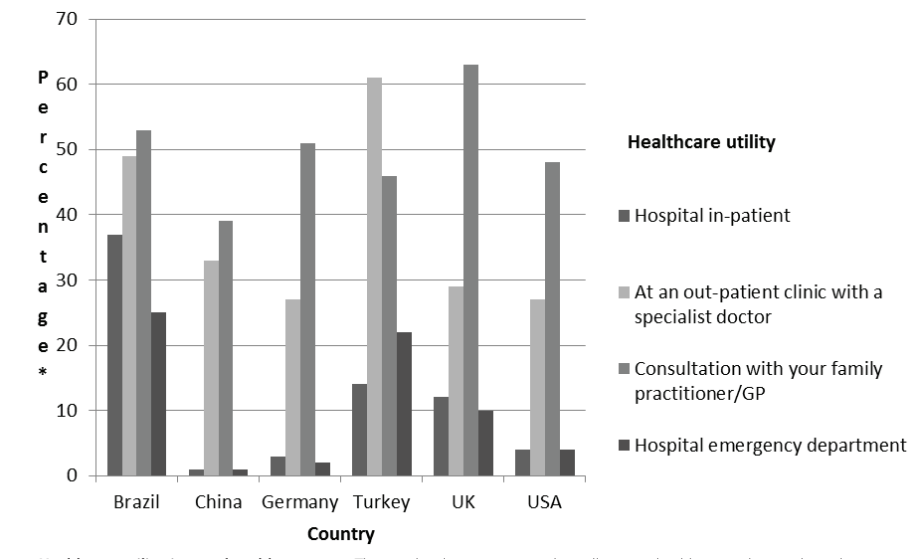
**Healthcare utilisation analysed by country**. The graphical representation here illustrates healthcare utilisation by utility type, and is presented based on a within-country analysis. *percentage of respondents utilising a healthcare resource within the last month. Respondents reported all utilities used within the last month.

Health care utilisation varied by country both by the type of service and the extent to which it was used [Figure 3]. The greatest proportion of health utilisation was in primary care however hospital inpatient care accounted for 68% of the total costs. Overall mean monthly cost per individual was $197 [£125], but this varied by country with China being the lowest [$6, £4] and UK being highest [$437, £277]. Total annual costs for the whole sample were estimated to be $5.74 m [£3.63 m;]. These estimates exclude the cost of pulmonary rehabilitation; although 12.3% of respondents had accessed that service in the previous month. It did not prove possible to attain a definition of pulmonary rehabilitation which could be applied consistently both within and between the countries in the sample.

Use of all types of healthcare resource increased with disease severity. For example in mild disease 34.3% of patients attended the GP and 4.5% required hospital in patient care whilst in those with severe disease the equivalent figures were 67.4% and 25%.

### Work productivity

Seventy per cent of respondents [n: 1690/2400] were not employed at the time of questioning. Twenty-six per cent of these respondents [n: 447] reported that they had given up work because of their COPD. The mean age of these early retirees was 58.3 years [range 45 - 67], of which 53% were female [n: 239/447]. Over half [52%] had moderate disease [n: 231/447], and 40% severe disease [n: 177/447]. Of those not in work who did not retire early due to their COPD [n: 1243], 52% were female and the mean age was 57.8 years.

The majority of those continuing to work were aged between 45-54 years [n: 447/710]. The WPAI scores suggest that COPD affected work productivity [Table 6] and the loss of productivity was more marked with advancing years. Within the group who were still working, impairment of regular activities outside of work is greater than overall work impairment. However, presenteeism was more common than absenteeism.

**Table 6:** Productivity [WPAI] and age

		Age range		
	45-54 years	55-64 years	65-67 years	
WPAI				Total
	% mean [CI]	% mean [CI]	% mean [CI]	
	*n*	*n*	*n*	*n*
Absenteeism	3.2%	6.5%	10.7%	4.7%
[% who missed work due	[2.0, 4.4]	[3.8, 9.3]	[0.3, 21.0]	[3.4, 5.9*]*
to COPD in the last week]	*n *= 411	*n *= 216	*n *= 32	*n *= 659
Presenteeism	8.8%	11.7%	14.9%	10.0%
[% who were affected in	[6.9, 10.7]	[8.4, 15.1]	[6.4, 23.3]	[8.4, 11.7]
the last week due to their	*n *= 447	*n *= 230	*n *= 33	*n *= 710
COPD]				
Regular activities	10.7%	15.4%	28.5%	13.0%
[% who were affected	[8.5, 12.9]	[11.7, 19.2]	[18.2, 38.8]	[11.1, 15.0 ]
within the last week due	*n *= 447	*n *= 230	*n *= 33	*n *= 710
their COPD]				

Disease severity also appeared to affect productivity at work although those with mild disease were less affected. However, a considerable increase in impairment was observed in those with moderate disease across all measures. Overall there was a considerable smaller percentage of people with severe disease still in active employment [see Table 7].

**Table 7:** Productivity [WPAI] and severity level

		Severity		
				
WPAI	Mild	Moderate	Severe	Total
	% mean [CI]	%mean [CI]	% mean [CI]	% mean [CI]
	*n*	*n*	*n*	*n*
Absenteeism	2.2%	8.3%	7.6%	4.6%
[% who missed work due	[1.2, 3.2]	[5.2, 11.5]	[2.1, 13.0]	[3.3, 5.9]
to COPD in the last week]	*n *= 383	*n *= 205	*n *= 54	*n *= 642
Presenteeism	5.2%	16.8%	18.9%	10%
[% who were affected in	[3.7, 6.7]	[12.6, 19.6]	[10.4, 27.3]	[8.3, 11.7]
the last week due to their	*n *= 405	*n *= 226	*n *= 62	*n = *693
COPD]				
Regular activities	5.9%	20.7%	32.3%	13.1%
[% who were affected	[4.1, 7.7]	[16.9, 24.4]	[22.8, 41.7]	[11.2, 15.0]
within the last week due	*n *= 405	*n *= 226	*n *= 62	*n = *693
their COPD]				

The average annual financial loss per patient from lost working hours was $880 [£556], totalling $596,760 [£376,412] for the cohort and the lifetime losses, $7,365 [£4,661]. This ranged considerably by country due to the differences in lost working hours and the wide variation in average salaries; with lifetime losses in China calculated at being the lowest at $678 and Turkey the highest at $20,805 [Table 8].

**Table 8:** Financial loss from reduced working hours

Country	N	Annual average loss	Lifetime average losses
		*[$] [se]	**[$] [se]
Brazil	151	827[235]	5849 [1572]
China	215	77 [24]	678 [182]
Germany	161	923 [368]	8099 [3181]
Turkey	55	2,014 [585]	20,805 [6486]
UK	64	2,176 [924]	18,153 [8690]
USA	31	1,805 [1,223]	11,205 [8459]
*Total Weighted*	*677*	*880*	*7,365*
average***			

*Average working hours lost × local average wage × $ppp

**Sum of annual income loss each year up to statutory retirement age discounted to present value at 3.5% per annum

*** Weighted average across whole sub-sample of 677 with work loss.

For those who retired prematurely due to COPD [n: 447], the average lifetime earnings losses were estimated to be $316,000 [£200,000] per individual. This equates to a total lifetime loss for this group of $141 m [£89.6 m].

Twelve percent [n: 84] of those in work had changed their employment to one which was either part time or less physically demanding. Twenty-eight percent [n: 201] of respondents in work were concerned that their condition would affect their career progression. Loss of income worried those both still in work and those not working. Forty-four percent [n: 314] of those still employed expressed concern about the consequences of COPD on their future earning capacity.37% [n: 896/2426] of all respondents reported that their total income had decreased as a consequence of their COPD, of which 80% [n: 719] indicated that this had a negative effect on their lifestyle.

Twenty-two percent [n: 534] of the sample required regular care or help from a family member, friend or neighbour [as opposed to a professional carer]. Of these, 289 [54%] needed this care either constantly or for part of every day.

The potential annual loss of income to carers was calculated as being $16,045 [£10,155]. However, if data are used only for carers of respondents who answered positively to the question 'has your need for care had an impact on the work activities of your carer' [39% n: 54] the annual loss amounted to less at $6,431[£4,070].

## Discussion

The aim of this study was to measure the personal, economic and societal impact of COPD on an international younger, working aged population. The results in each country suggested a high level of impact of COPD in terms of personal quality of life, patient and carer commitments to work and utilisation of health care resources. Data varied between countries but many consistent patterns emerge overall.

The economic impact was revealed by the high percentage of people who had been forced to stop working due to their COPD [~40% n: 447/1157]. This supports the existing literature which has shown that approximately one in five people are likely to retire prematurely due to their COPD [20,33,34]. We conducted a sensitivity analysis which assumed a standard retirement age of 67 [rather than 65]; this raised the estimate of productivity loss from early retirement due to COPD to over $160 m [£101 m]. If, as has been proposed in many countries, retirement age is raised [35], the costs from premature retirement will rise.

Of those reporting to have retired early due to their COPD, 40% [n: 177/447] had severe disease compared to 9% [n: 62/710] with severe disease who remained in work. Disease severity at the point of retirement was unknown, however, 64% [n: 284] of retirees had retired at least 4 years before the study so their disease severity may have worsened. It is therefore not possible to state conclusively that disease severity is the main cause of early retirement. The ability to remain in work may well be dependent on other factors such as the type of employment and the flexibility of the employer, as some people with moderate disease do continue to remain actively employed [32%, n: 226/710] and yet many do not [52%, n: 231/447]. This would be worthy of further research and may help to find some of the solutions to keeping people with COPD in active work for longer.

There are several reasons, apart from their COPD, for a large number of the survey population not being in work [n: 1243] although still of working age. In this group there are likely to be people who had; actively decided not to work, already retired, were unemployed and those who had retired early for another personal or health reason. The ability to remain in active employment with COPD was an issue for all respondents from every country, who had reported concerns about their future earning capacity. As a result, they felt unable to maintain their previous lifestyles and had difficulty planning ahead in terms of financial commitments. Other respondents reported continuing to work despite their COPD, although it was noticeable that those with more severe disease were less likely to remain in employment. Those with moderate and severe disease were more severely affected in terms of productivity than those with mild disease. There were some inter-country differences, perhaps explained by differences in welfare and benefits systems. The absence of any or limited paid sick leave may pressurise individuals to decide between caring for their deteriorating health or potentially losing jobs and income. Therefore, the costs of presenteeism may extend beyond lost productivity, should COPD patients decide to continue working.

The survey was conducted during the summer months for all countries in the Northern Hemisphere [although this period was wintertime for Brazil]. COPD exacerbations are more common in the winter months [36], so there may have been some under reporting of the extent of the effects of COPD on work productivity and health care utilisation.

Many respondents reported difficulty maintaining their activity levels outside of work. The number of those remaining employed, and levels of presenteeism, suggest some significant efforts are made in terms of remaining employed. It is possible that as a result of the effort to remain in work, respondents were making sacrifices in terms of their capacity to maintain social activities. Many family members also reported having to give up work to care for relatives. As COPD predominately affects people on lower incomes [37] this potentially has serious consequences for families experiencing a dual loss of income from patient and carer employment limitations. When coupled with lack of clarity about prognosis and anticipated morbidity, patients with COPD and their families face a future of uncertainty [38].

In our study, there was considerable variation across countries in the volume, costs and type of health care utilisation, reflecting differing health systems and patterns of disease severity. Our findings, however, suggest that COPD patients are heavy users of health care and that the costs associated with this are high with an average healthcare utilisation cost of $2,364 [£1,500] per annum per capita leading to a total amount for this cohort of $5.74 m [£3.63 m;] per annum. Other studies have estimated the direct cost of COPD care to be significantly less, around $1234-1823 [£781-1154] per patient [39]. As in previous studies, the greatest costs were derived from hospital inpatient care [26,40]; however in our study far more patients reported having visited their family practitioner at a substantially lower unit cost. Whilst it is difficult to draw conclusions between studies due to differences in measurement, healthcare utilisation costs are high across all studies. Although COPD is currently under-diagnosed [3], prevalence is increasing due to increasing numbers of people exposed to risk factors, the aging population, and earlier detection. Hence the total cost of healthcare for COPD is likely to increase, potentially placing further burden on primary care and hospital care. Whilst further research is needed to establish the complete picture in terms of cost effectiveness, skilled multidisciplinary teams in primary care may help to reduce COPD hospitalization rates [41]. A recent survey within UK general practice however suggested 52% of respondents lacked the appropriate training to manage people with COPD effectively [42]. As the majority of COPD care is delivered within primary care, this highlights the need for investment in sustained educational interventions that have been shown to improve the quality of life of people with long term conditions [43].

The impact of COPD on quality of life observed in our study has been previously reported using a variety of both generic and disease specific instruments [44]. Similarly, and in line with previous research [45], health- related quality of life [HRQOL] measured by EQ-5D was worse for those with more severe disease and higher numbers of comorbidities. The extent of comorbidity in this younger study population was considerable and would appear to be higher than reported before [48-52%] [17,34]. From the results of this survey it is not known the extent to which comorbidity affected the ability to continue in active employment; however this would worthy of further investigation. It is recommended that patients with airflow limitation should routinely undergo comprehensive clinical assessments to identify co-morbid disease [46]. It would appear this is particularly important in younger patients, as earlier and more aggressive interventions may keep individuals in employment for longer.

Published demographic data suggests that COPD is currently more prevalent in men [12] There were similar proportions of women and men in this study. This may reflect the gender differences in survey response rates in general [47]. Our study may also be reflecting the changing trends in female smoking behaviour, and that impact on COPD prevalence [48].

It is concerning that almost two thirds of the participants continued to smoke. As smoking cessation is currently the only known intervention to alter disease progression [49]; workplace programmes may be helpful to those still in employment.

It was not our intention to make country comparisons, however the results from China are worthy of note due to the apparent differences between China and the other countries in all measured parameters. The Chinese participants, the majority of whom were male [n: 283, 71%], reported milder disease [as measured by MRC score], higher quality of life, minimal loss of productivity and less use of health care services yet the Chinese data contained the highest number of current smokers [n: 329, 83%] compared to the rest of the cohort 59% [n: 1366]. We can surmise from the size of the cohort and the chosen methodology that these differences may be culturally specific rather than a reflection of physical effects of the disease. The concept of 'face' or 'mianzi', whereby the social standing of an individual is in relation to others, is part of Chinese belief systems. Losing face is something to be avoided and can give rise to distress caused by shame. It is possible that our questions relating to disease severity, comorbidities and work were influenced by these beliefs, resulting in the apparent differences between the Chinese data and the rest of the cohort.

The main strength of this study is that the large dataset was collected from similar sized samples of the population in six countries with differing economic, social and demographic characteristics thus presenting a wide international perspective on the disease impact. However this also gives rise to some of the limitations as the study population cannot be seen as representative of any larger domain, and the value of pooled analyses is limited. The advantage of the approach is that a by-country analysis identifies those aspects of COPD, and its impact on patients, which are common to a widely differing set of countries, and those which are limited to certain societies. The breadth of the data collected serves to indicate some of the possible reasons for differences in the pattern of the disease and its impact between countries. Consistency in many qualitative relationships, for example between COPD severity and quality of life, can be seen across all countries. Whilst pooled data analysis is not appropriate, to the extent that the sub-samples are representative of COPD patients in each country, estimates of aggregate national burdens may be made.

One major limitation in this study is the fact that there were differences in the methods of selection of the study population, recruitment and data collection across countries. This may have resulted in the observed differences in the characteristics of the patients; for example, patients recruited from local patients' groups [UK] may have had more severe disease than those recruited randomly from geographical regions [China, Turkey]. The significant heterogeneity of disease severity between countries [data not shown] prevents us from pooling the data or making cross-country comparisons. However, these data are still valuable to illustrate the global picture of the burden of COPD.

Where available we used validated questionnaires to quantify the burden of COPD in a working age population. However, there were some aspects of COPD for which these questionnaires were not available. This resulted in the development of a small number of specific, non-validated questions. These questions, despite being piloted with COPD patients and revised to minimise ambiguities and errors, may not reflect COPD morbidity with the same degree of reliability as the validated questionnaires.

Although we used criterion which would identify participants with COPD as accurately as possible, the diagnosis and reported severity was not verified by spirometry. The survey was subjective and retrospective in nature, relying on patients' recollection of the impact of COPD over varying time periods; this may have resulted in either over or underreporting of its impact [50]. The method of interview [telephone versus face-to-face] may also have had an impact on the way the questions were answered with telephone respondents having the potential to underplay their symptoms in an attempt to reduce social desirability bias [51]. Many other differences observed in this study would reflect the demographic pattern of disease, occupational contexts, economic drivers and access and affordability of health care.

Further prospective studies are required to investigate the impact of COPD on people in work, the precipitating factors for early retirement, and identify interventions that may keep people actively employed.

## Conclusions

This survey demonstrates that COPD has a marked effect on the working age population. The cost of illness is possibly more extensive than has previously been recognised [5,12].

Significant societal benefits could be achieved if COPD were diagnosed earlier and managed appropriately as this may enable individuals to optimise their ability to remain in active employment. Health professionals should ensure they have the skills to recognise the considerable burden COPD incurs, and the knowledge to treat sufferers effectively. This in turn should be supported by a commitment to invest in appropriate and accessible health services, including preventative health and to develop workplace strategies which enable people with COPD to remain in active employment for longer.

While some issues related to the study design may limit the scope for generalisation to all COPD populations, we hope the issues raised will increase the focus of attention of the impact of this condition on the younger populations.

## Competing interests

MF has received monies on behalf of Education for Health for activities including: advisory boards and speaking/chairing meetings for AstraZeneca, GlaxoSmithKline, Novartis, Boehringer Ingelheim, Nycomed, and NAPP Laboratories. The organisation has also received research funding and/or educational scholarship funding from Novartis, GlaxoSmithKline and AstraZeneca. MF has been supported to attend international meetings by Boehringer Ingelheim, GlaxoSmithKline and AstraZeneca. JU has no competing interests. JTF has no competing interests. ASB is a member of Advisory Boards for Merck, GSK, Novartis, Sepracor and has received unrestricted educational funds for the BOLD Operations Center through the Kaiser Permanente Center for Health Research in Portland, Oregon, USA. CJ has received consulting fees, fees for education provision and for attending advisory boards from AstraZeneca, GlaxoSmithKline, Novartis, Nycomed and Pfizer P/L. CJ is employed by the Woolcock Institute of Medical Research which receives research support from GlaxoSmithKline and educational funding and support from AstraZeneca. This helps support research and administrative programs. NB has received lecture fees and consultancy from GlaxoSmithKline [GSK] Astra Zeneca [AZ], Merck Sharp and Dohme, Nycomed, Forest Pharmaceuticals, Almirall and Novartis. For research purposes, GSK, AZ, Almirall and Novartis have provided funding. JH is Director of York Health Economics Consortium which receives grants for research from pharmaceutical companies including some with interests in respiratory care. YHEC staff do not undertake personal consultancy. TVDM has no competing interests. JW has a diagnosis of COPD. JW is CEO of 3 COPD not-for-profit organizations. JW donates all honorariums, fees for speaking, etc. to the COPD Foundation or the Alpha-1 Foundation and raises funds from sources, except tobacco, for research and education. PWJ has no competing interests. SW has received lecturing and/or consultancy fees from Astra Zeneca, GlaxoSmithKline and Novartis. She has been supported to attend international conferences by GlaxoSmithKline, TEVA and Novartis.

## Authors' contributions

MJF conceived of the study, participated in the design of the study, interpretation of the data and drafting of the manuscript. JU participated in the design and co-ordination of the study, interpretation of the data and drafting of the manuscript. JT-F participated in the design and co-ordination of the study, interpretation of the data and drafting of the manuscript. SAB participated in the design of the study, interpretation of the data and critical revision of the manuscript. CJ participated in the design of the study, interpretation of the data and critical revision of the manuscript. JH participated in the design of the study, statistical analysis, technical support, interpretation of data and revisions to the manuscript. NB participated in the interpretation of the data and critical revision of the manuscript. TVDM participated in the design of the study, interpretation of the data and critical revision of the manuscript. JWW participated in the interpretation of the data and critical revision of the manuscript. PJ participated in the design of the study, interpretation of the data and critical revision of the manuscript. SW participated in the conceptualisation and design of the study and drafting of the manuscript. All authors have read and approved the final manuscript.

## Pre-publication history

The pre-publication history for this paper can be accessed here:

http://www.biomedcentral.com/1471-2458/11/612/prepub
